# Role of Nanotechnology and Their Perspectives in the Treatment of Kidney Diseases

**DOI:** 10.3389/fgene.2021.817974

**Published:** 2022-01-05

**Authors:** J. P. Jose Merlin, Xiaogang Li

**Affiliations:** ^1^ Department of Internal Medicine, Mayo Clinic, Rochester, MN, United States; ^2^ Department of Biochemistry and Molecular Biology, Mayo Clinic, Rochester, MN, United States

**Keywords:** nanotechnology, nanoparticles, nanomedicine, drug delivery, kidney disease

## Abstract

Nanoparticles (NPs) are differing in particle size, charge, shape, and compatibility of targeting ligands, which are linked to improved pharmacologic characteristics, targetability, and bioavailability. Researchers are now tasked with developing a solution for enhanced renal treatment that is free of side effects and delivers the medicine to the active spot. A growing number of nano-based medication delivery devices are being used to treat renal disorders. Kidney disease management and treatment are currently causing a substantial global burden. Renal problems are multistep processes involving the accumulation of a wide range of molecular and genetic alterations that have been related to a variety of kidney diseases. Renal filtration is a key channel for drug elimination in the kidney, as well as a burgeoning topic of nanomedicine. Although the use of nanotechnology in the treatment of renal illnesses is still in its early phases, it offers a lot of potentials. In this review, we summarized the properties of the kidney and characteristics of drug delivery systems, which affect a drug’s ability should focus on the kidney and highlight the possibilities, problems, and opportunities.

## Introduction

The term nanotechnology was created by Professor N. Taniguchi for the first time in 1974. Drexler designed the term “nanotechnology” and published it in his book “Vehicles of Creation: The Arrival of the Nanotechnology Era” in 1986 (Bayda et al., 2020). Nanoscience is the only field that can collaborate with traditional fields like health discipline, biological chemistry, medicinal remedies, perception, nutriment, maquillage, auto electronic, commodity, and even designed to find new qualities of matter. Nowadays, a well-constructed fusion of applied science has been used to address difficulties in the specialty of biomedical sciences by generating more effective medical management, nanomedicines, and remedial (Bayda et al., 2020; Sahu et al., 2021). The influence of nanotechnology on humans and animals may open new avenues for research and transformation in the field of health science, and it has become an urgent topic for consideration as a therapeutic tool (Sichert et al., 2015). Nanotechnology is a shadowy multidisciplinary field that was created to engineer biological matter like atoms, molecules, and supramolecules (Sichert et al., 2015; Sahu et al., 2021). Nanoparticles (NPs) are one-dimensional objects that are 1–100 nm in size. Nanotechnology refers to the development and production of NP materials on an atomic or molecular size (Ma et al., 2020). Both organic and inorganic materials can be used to make NPs. Organic NPs include polymeric, carbon, liposomes, and dendrimer-based NPs. Quantum dots (qdots) and magnetic iron oxide particles are examples of inorganic NPs (Sung and Yang, 2015). The ability to manipulate and tailor the physiochemistry of materials by the nanometer scale, where molecular reactions occur, which advance to a slew of possibilities in the medicines, including observation of biomarkers in prior stage, focusing cells and tissues, advanced in drug delivery, evaluating end-stage disease, and more (Ratner, 2019). In medical nanotechnology, NPs are used in the depiction, assembly, dominance, and application of curative medications and implements (Contera et al., 2020). In this review, we are focusing on the challenges and perspectives related to nanotechnology in kidney diseases.

Kidney disorders are multistep processes involving the accumulation of a variety of molecular changes. The cellular function of kidney cells and their surroundings is affected by these molecular alterations. Many genetic changes, such as mutations, loss of heterozygosity, deletions, insertions, and aneuploidy, have been linked to various kidney illnesses (Sadikovic et al., 2008; Li et al., 2019). In terms of kidney function progression, kidney diseases are separated into acute kidney injury (AKI), and chronic kidney disease (CKD). AKI is established as a rise of creatinine level in the blood about 0.3 mg/dl in less than 48 h and increased to 1.5 times in less than 7 days, and urine volume of 0.5 ml/kg/h is more than 6 h. Hypovolemia and urinary blockage, drug poisoning is the most common causes. A determined irregularity of structure and function of the kidney (e.g., albuminuria >30 mg per 24 h or glomerular filtration rate (GFR) 60 ml/min/1.73 m^2^) for more than 3 months is characterized as CKD (Luo and Grams, 2020). The most prevalent causes of CKD include diabetes, hypertension, and primary glomerular disease. In terms of definition, causation, and treatment approaches, they are very different. CKD is a global health concern that affects more than 10% of the population, with a higher prevalence among the elderly (Luo and Grams, 2020). Patients with CKD usually endure a slew of problems and negative outcomes, putting a significant financial strain on both the people affected and society as a whole (Saran et al., 2019). Autosomal dominant polycystic kidney disease (ADPKD) is a typical hereditary dysfunction in humans with a diagnosed prevalence of about 4 in 10,000 implying that approximately 140,000 people in the United States are affected (Ters et al., 2020). The aberrant, cell-autonomous proliferation of cyst epithelial cells in ADPKD is linked to cellular dedifferentiation and overexpression of multiple proliferative factors, and it resembles a cancerous process (Mochizuki et al., 1996). As a result, early detection of CKD and quick disease prevention are becoming a public health priority. There is evidence that nanoparticles might be involved in the creation of renal calculus, polycystic kidney disease, gallstones, and gallbladder inflammation, prostatitis, calciphylaxis, and ovarian cancer, among other diseases and degenerative processes (Grantham, 1990).

Kidney disease is a global issue that affects about 750 million people worldwide, with the severity of the condition varying greatly. The rate of kidney disease and the availability of care for it are largely determined by social, cultural, and political variables, all of which contribute to a major imbalance (Crews et al., 2019). Furthermore, guidelines have been recommended that kidney disease could be recognized as a life-threatening disorder that affects people who require nephrologist’s care, as well as a common disorder with varying severity that requires a concerted public health approach for prevention, early detection, and management (Rettig et al., 2008; Levey and Coresh, 2012). Guidelines have a significant impact on clinical practice, research, and public health, but they have also sparked debate (Eckardt et al., 2009). Complement activation is imbalanced in the kidneys, which have a conspicuous, compartment-dividing membrane, and the glomerular basement membrane, which lacks complement regulators and might be at risk to attack when the cell layer is damaged (Ricklin et al., 2018). More factors, such as high complement component concentrations, pH changes, and disruption or vulnerability of the glycocalyx lining, may increase the susceptibility of the kidney to complement-induced injury (Thurman, 2015). On the other hand, complement-mediated kidney diseases are caused by a wide range of pathogenic mechanisms (Crews et al., 2019). Nanoparticles are currently being used by researchers to develop novel therapeutic medications that can reduce side effects while increasing efficacy. Nanoparticles can also combine numerous functions such as therapeutic and diagnostic capabilities, on a single nanoparticle platform (Chin et al., 2020).

## Synthesis and Characteristics of Nanoparticles

Drug delivery systems using NPs have been developed in recent decades to solve the major problems of ocular drug administration, resulting in a safe and effective system that delivers the medicine to the appropriate region (Qamar et al., 2019). Development of various nanotechnology-based nanomedicines with different novel systems, including liposomes (Urquhart and Eriksen, 2019), solid lipid NPs (Patil et al., 2019), nanostructured lipid carriers (NLA) (Ozdemir et al., 2019), dendrimers (Villanueva et al., 2016), polymeric NPs (Mandal et al., 2017), inorganic NPs, microemulsion, nanosuspension, nanoemulsion, and noisome, which results in increased retention time, the solubility of hydrophobic drugs, bioavailability, enhanced drug penetration, and target specific sites. The encapsulation of the desired medicine in a nanoparticle also helps to protect it from degradation (Qamar et al., 2019).

### Synthesis of Nanoparticles

The most prevalent and industrially used strategies for producing polymer nanoparticles are heterophase polymerization procedures (Demirdogen, 2019). The most common heterogeneous polymerization process is emulsion polymerization. Hydrophobic monomers are composite in an aqueous phase, which is trailed by polymerization in a direct process and oil-in-water emulsions (Jenjob et al., 2018). Surfactants with electrostatic and steric stabilization are commonly used to secure colloidal systems to prevent coagulation throughout chemical reactions (Kurozuka et al., 2017). Submicron polymer particles are produced via emulsion polymerization, with size ranges from 50 to 500 nm (Thickett and Gilbert, 2007). A classic size of the particles includes between 1 and 10,000 macromolecules, each of which is made up of 100–106 monomer units (Chern, 2008). Hydrophilic monomers like acrylamide, acrylic acid, and methacrylic acid are combined and change integrity in a continuous phase (Yamak, 2013). Solid particles from Pickering emulsions, which are emulsions of particle-stabilized emulsions with long-term stability (Jenjob et al., 2019), can be used to stabilize emulsions. Because of their greater stability against coalescence, colloidal particle-stabilized Pickering emulsions have obtained a lot of scrutiny in the current period. Yang et al. have been evaluated the usage of numerous solid particles as Pickering emulsifiers, including silica, clay, magnetic NPs, nanotubes, and as well as their applications in diverse domains (Piao et al., 2015). There are several synthesis methods are used to prepare nanoparticles, which are summarized in Table 1.

**TABLE 1:** Summarize the various synthesis methods of nanoparticles.

Synthesis method	References
Double emulsion solvent evaporation	Bitar et al. (2015)
Single emulsion solvent evaporation	Khalil et al. (2013)
Nanoprecipitation method	Han et al. (2013)
Emulsion diffusion method	Campos et al. (2013)
Emulsion polymerization	Wu et al. (2011)
Microemulsion Technique	Destree et al. (2007)
Salting out	Miller et al. (1988)
High Pressure Homogenization	Shi et al. (2011)
Coacervation method	Wieland-Berghausen et al. (2002)
Phase Inversion Temperature Method	Saberi and McClements, (2015)
Solvent Injection Method	Yegin et al. (2006)
Solvent displacement method	Muhamad et al. (2020)
Solid-in-oil-in-water method	Sabaeifard et al. (2016)
Chemical co-precipitation method	(Wang et al., 2018)
Ionic gelation method	Pandey et al. (2019)
Fabrication method	Ibrahim et al. (2020)

### Characteristics of Nanoparticle-Based Drug Delivery

The most essential aspects of nanoparticles are their particle size and size distribution, which determine the delivery methods such as *in vivo* dispersion, biological destiny, toxicity, and their targeting capabilities. They also have an impact on drug loading, drug release, and nanoparticle stability. Nanoparticles have a lot of advantages over microparticles (Yang et al., 2017). One of the most difficult challenges is the characterization of synthesized NPs. The techniques most widely employed to analyze biosynthetic NPs include dynamic light scattering (DLS), transmission electron microscopy (TEM), ultraviolet-visible (UV-vis) spectroscopy, Fourier-transform infrared spectroscopy (FT-IR), and X-ray diffraction (XRD).

#### Size Determination

For quick concealing of quality control and polydispersity of the samples with low dispersity, DLS is advised, which can be utilized for high-resolution characterization of complicated samples when combined with other technology. During the formulation development, AF4-MALS/DLS has been reported as an image sizing technique (Singh and Lelard, 2009). Number, volume, or mass-based size distributions, polydispersity, and particle concentrations should also be recorded, according to the U.S. Food and Drug Administration (FDA)’s draught advice on nanomaterial-containing formulations (Caputo et al., 2019). The intensity-based size distribution might be used to construct the character and accumulation-based size distributions by using a specific particle shape model, which would otherwise introduce huge inaccuracies when big (Rh > 200 nm) particles are present (U.S. FDA, 2017). As a result, alternative approaches, such as both DLS and microscopy, are advised for reporting intensity- and number-based size distributions, respectively (Wyatt and Weida, 2003). Furthermore, Filipe et al. conducted comprehensive comparative research using DLS and nanoparticle tracking analysis (NTA) to measure particles size ranging 60–1,000 nm and found that NTA recognized polydispersity and big aggregates are better than DLS (Fan et al., 2021).

#### Surface Charge

The particle’s charge profile in relation to a diffusive layer is depicted by the zeta potential, which may be estimated from the electrophoretic potency of particles are measured by phase analysis light scattering (PALS) (Filipe et al., 2010). In order to obtain precise measurements, important medium variables such as nature of the phase, refractive index, viscosity, and temperature, must be pre-decided. Indeed, multiple studies have found that the zeta potential of liposomes is affected by the media’s pH, temperature, and ionic strength (Smith et al., 2017), protein adsorption (Fatouros et al., 2005), and encapsulation of charged active pharmaceutical ingredients (APIs) (Matsumura et al., 1994). The molar % of the ionic lipids included in the liposome had a linear connection with the zeta potential (Filipe et al., 2010), and a value of -30 mV or >30 mV might be generally maintained by sufficient inter-particle repulsion and stable particle suspension (Matos et al., 2004). Even though the polyethylene glycol (PEGylated) lipid is introduced at 0.2 mol percent, PEG modification can protect lipid charges and stabilize liposomes (Filipe et al., 2010). The fluorescence labeling could be able to determine the entire surface potential of liposomes (Wyatt and Weida, 2003).

#### Morphology

Several Transmission electron microscopy (TEM) techniques, including negative stain (reagent used are uranyl acetate and uranyl formate), freeze structure, and cryogenic microscopy, are used to detect the size and morphology of the particles (Honary and Zahir, 2013). Recently, cryo-TEM is used to develop images directly after vitrification, giving focus morphology and comprehensive structural facts on lipid layers and encapsulation appliances. Because of the disruptive operations for scanning electron microscopy (SEM), is used an electron beam to scan over the surface of the sample, which used after sample preparation, such as drying, fixing, and imaging under high vacuum are not typically employed for imaging of lipid particles (Severs, 2007). Furthermore, atomic force microscopy (AFM) might be utilized to analyze the three-dimensional structure of liposomes by measuring interactions between the sample surface and a probing tip. Robson et al. compiled a detailed comparison of various imaging techniques for liposomes (Mohammed et al., 2004).

#### Ultraviolet-Visible Spectroscopy

During synthesis, a distinct color change is frequently used as a visual signal of the creation of NPs. Depending on the NPs size, nanoscale gold exhibits a deep purple color. As a result, UV–vis, a general and low-cost method of confirming the existence of NPs. A plasmon resonance exists on every metal surface that corresponds to a certain visible wavelength (Robson et al., 2018). The morphology of particles includes the type of capping agents and the reflecting index of the surrounding medium all influence the position of a resonance band (Aiken and Finke, 1999). Du et al. discovered a faint absorption peak at 535 nm, which corresponded to the creation of gold NPs (AuNPs) in an E. coli solution treated with gold ions for 54 h (Du et al., 2007). After another 120 h of incubation with the same ion concentration is the significantly broader peak, indicating 20–30 nm gold colloids, and particle shape can be seen in UV–vis. He et al. discovered a considerable shift in the absorbance spectrum as a basis of particle structure (He et al., 2008). UV–vis reacted with a characteristic wavelength of 520–550 nm (ruby red), with NPs size 10–20 nm at concentrations as low as 0.25 mM. In the region of 500–900 nm, however, the absorption peak got wide-ranging with the concentration of 0.5 mM. (gray-blue). The development of gold nanowires 50–60 nm in length was blamed for the modifications. For silver NPs (AgNPs), the absorption peak was at 380 nm (deep brown) (Kalathil et al., 2011). Another study discovered a prominent absorption peak for ZnS NPs encapsulated by rhamnolipids at 340 nm (blue) with cationic surfactant, cetyltrimethylammonium bromide (CTAB) is utilized as capping agent, the absorption peak moved to 294 nm (Hazra et al., 2013).

#### Fourier Transform Infrared Spectroscopy

Fourier transform infrared spectroscopy (FTIR) analysis is used extensively to identify binding molecules or functional groups on the surface of NPs generated in a biological matrix. Infrared radiation with a wavelength spanning from 4,000 to 400 cm−1 can be absorbed by biomolecules in the matrix (Davis and Mauer, 2010). In a bacterial matrix of E. coli, a weaker wavelength of 988 cm−1 was observed with rhamnose and a stronger wavelength of 1,385 cm−1 relating to the carboxyl groups, which showed aldehyde group oxidation to carboxyl groups, which led to AgNPs immobilization (Kang et al., 2014). FTIR has been employed by several researchers for similar purposes, because of the complicated sample architectures, the application of FTIR is limited. The complex composition of a biological matrix, combined with the intensity of overlying of infrared absorption bands limits the application of this approach to NPs characterization (Rieppo et al., 2012).

#### X-Ray Diffraction

The morphology of NPs can be determined by X-ray diffraction (XRD). To establish the crystal structure of biologically generated NPs, one or other approaches are frequently used. Bragg reflections matching to distinct crystal planes of NPs can be seen on XRD (Kalathil et al., 2011; Pat-Espadas et al., 2014). Selected area electron diffraction (SAED), on the other hand, can be used in conjunction with TEM imaging, which provides a comparable level of study as XRD (Kalathil et al., 2011).

#### Stability of Nanoparticles

NPs are the factor-dependent dispersion stability with solid core surrounded by suitable surface chemical microenvironment, which influenced the size, polarity, molecules on the surfaces, solvent polarity, surfactants, solvents, and other factors of NPs (Xu et al., 2018). For specific solvents, the polarity and quantity of molecules coated on the surface determine the dispersion stability of NPs. pH, ion concentration, temperature, and storage period are some of the other parameters that affect NPs dispersion stability (Piella et al., 2016). The dispersion stability of NPs is dispersed in the liquid phase has deteriorated, resulting in irregular NP aggregates. The two NP aggregates are homo-aggregation and hetero-aggregation (Huynh et al., 2012). The aggregation of NPs is significant in a variety of applications, including filtration, flotation, medicine, and water purification (Yu and Borkovec, 2002), and the composition, shape, size, and surface charge of NPs vary (Lopez-Lopez et al., 2006). Thermal activation could accelerate and exacerbate the processes of diffusion, relaxation, grain development, and homogenization, resulting in partial or total structural damage of the NPs and performance degradation of the nanodevices (Andrievski, 2003; Andrievski, 2014). When bulk materials are reduced to the nanoscale, the melting point drops dramatically, resulting in poor thermal stability, which severely limits the use of NPs in high-temperature processes. The thermal agitation of atoms during heating results in two common processes: sintering and fusion (Buffat and Borel, 1976; Satterfield, 1991).

### Encapsulation Efficiency and Drug Loading Capacity

Encapsulation is such a common pharmaceutics technique and is unavoidable to overcome a substantial volume of literature describing encapsulated formulations, such as nanomedicines and drug delivery systems (DDSs) (Brayden and Alonso, 2016). The public’s enthusiasm for nanomedicine had heightened the desire to participate in this formulation race. Nowadays, anecdotes and cartoons are abounded to persuade academia, industry, and the general public that nanotech goods will perform miracles and deliver a brighter future for humanity, and it is debatable of its expectations (Park, 2017). Even though it would rather give a critique of the reasons presented to explain the few efficiency phrases, such as entrapment, loading, and encapsulation, which are now widely encountered in encapsulation literature. Encapsulation also has additional loading-related terminology, such as loading efficiency and effective drug loading (Sun et al., 2015). Drug encapsulation efficiency within lipid or polymer particles is low, especially for water-insoluble medicines that must be linked with the vesicle membrane when encapsulated in liposomes (De Cock et al., 2010). Avoiding these phrases while distributing experimental findings is deviating from the norm these days. Entrapment and encapsulation efficiency are used interchangeably in most circumstances because they are calculated using a mathematical equation (Bhattacharjee, 2018).

Therefore, drug loading capacity and encapsulation/entrapment efficiency are calculated using the equations:

$$Drug\;Loading\;Capacity\left(\%\right)=\frac{Total\;drug\;encapsulated}{Total\;nanocarrier\;used}\times 100$$

$$Encapsulation/Entrapment\;efficiency\left(\%\right)=\frac{\left(Total\;drug-loaded-Total\;drug\;encapsulated\right)}{Total\;drug-loaded}\times 100$$

### *In Vitro* Drug Release

*In vitro,* drug release investigations using nanoparticle formulations are more popular and adaptable with a dialysis membrane (DM) than with sampling and separation (SS). Regular dialysis and reverse dialysis are the most common DM treatments. The nanoparticle formulations are held inside the regular dialysis, allowing for simultaneous release and separation of the released API. The dialysis membrane, volume ratios between the sample and release media, and agitation settings are all important characteristics for this procedure (Xu et al., 2012). Unstable particles may collect and clog the membrane, particularly during long-term studies, or the released medication may be adsorbed on the membrane, causing drug release profiles to be underestimated in both cases (Yu et al., 2019). The continuous flow (CF) technique makes use of the United States Pharmacopeia (USP) IV (4) apparatus to circulate and modify the release media in a dynamic manner. Regular dialysis could be utilized to hold nanoparticle formulations on top of the glass beads in the sample chamber for usage with nanoparticle formulations. The combination DM-CF strategy better-distinguished dexamethasone release profiles from liposomes with varied lipid compositions than the traditional dialysis sac (DS) method (D’Souza, 2014).

Although drug release can be measured using a variety of ways, the main purpose of all these tests is to predict formulation pharmacokinetics *in-vivo* using *in-vitro-in-vivo* correlations (IVIVC), hence reducing the burden of research for generic product development. It would be preferable to have a linear or non-linear point-to-point correspondence between *in-vitro* and *in-vivo* release kinetics (Bhardwaj and Burgess, 2010). Therefore, the equation of *in vitro* drug release is:

$$In\;vitro\;drug\;release\left(\%\right)=\frac{Total\;drug\;released}{Total\;drug-loaded}\times 100$$

### Biodistribution of Nanoparticles

NPs are only biodistributed after injection, and they are carried by the bloodstream and distributed throughout organs and other tissue systems. During blood transfer, the nanoparticles are bound with serum proteins termed opsonins (Mirshafiee et al., 2016; Wei et al., 2018). Due to macrophages, the injected doses may result in a significant reduction in nanoparticle removal. The capability of lone circulation in the formation of nanoparticles could reduce the serum protein binding (Li and Huang, 2008). Glomerular filtration and tubular secretion are the processes of renal excretion (Krediet, 2017). The wall of the glomerular capillary has an intrinsic permeability property, which can filter substances such as lesser than 5.5 nm size or proteins weighing less than 3 KDa (Liu et al., 2013). Normally, the NP’s charge, shape, and size lesser than 100 nm could be easily filtered (Liu et al., 2013). The spleen is a big lymphoid organ with high vascularity, which produces lymphocytes by storing blood, disintegrating old blood cells, and filtering foreign materials out of the blood (Hoshyar et al., 2016). According to research, the size of 150 nm with spherical shape and larger particles with 200–250 nm are especially susceptible to filtration at the spleen’s interendothelial cell slits (Moghimi et al., 2012). The liver has several sinusoidal endothelial cells (LSECs), which are highly specialized endothelial cells that serve as a permeable barrier between blood cells and hepatocytes/hepatic stellate cells (Hang et al., 2012). Fenestrae allows nanoparticles to move from the sinusoidal lumen to the surfaces of hepatocytes, which results in increased absorption and retention in the liver. According to a study, the diameter of fenestrae in persons without liver disease is 107 ± 1.5 nm (Wisse et al., 2008). Another study found that NPs can be eliminated by the liver hepatobiliary system, which enters the bile duct via the bile canaliculi, gather inside the gallbladder close to the common bile duct, and then be expelled into the duodenum (Zhang et al., 2016). The enhanced permeability and retention (EPR) effect allows for a wide spectrum of NPs movement, which is dependent on their fenestrations and the lack of good lymphatic drainage for drugs and NPs accumulation (Orbay et al., 2016). Furthermore, endothelial cells use an antibody- and caveolae-dependent transcytosis pathway to transport particles from the blood into tumors, and the size of particles transferred into tumors is limited to 50–100 nm (Wang, 2014). In addition, the elimination of NPs has been documented in many different organs, rather than blood, kidneys, liver, and targeted tissues, including the NPs circulation station, capillary vessel borders in the lung, which may detain some NPs larger than 1,000 nm (Moghimi et al., 2012). The clearance of nanoparticles from the bloodstream is also aided by lymph nodes and skin (Larraneta et al., 2016). Most target receptors lack small-molecule ligands that engage strongly and specifically with their extracellular domains. The lead compound for small molecule targeting ligand development is a natural substrate, a small molecule inhibitor, or a transition state analog of the target receptors (Friedman et al., 2013). Synthetic small molecule targeting ligands, which are unable to generate small molecules, bind with high affinity and specificity to the targeted extracellular domains. Multivalent targeting, which is utilized to boost the bonding strength between two molecules, is a potential (Friedman et al., 2013).

## Applications of Nanoparticles in Biomedical Research

Nanotechnology is a major interdisciplinary field, which combines biology, chemistry, medicine, and engineering to promote and develop effective bio-medical tools. These approaches provide a new perspective and dimension to clinical practice and surgery. Nanomedicine incorporates a remarkable clash on drug delivery systems, tissue regeneration programs, medical diagnostics, detection, and introducing new materials (Chandarana et al., 2018). The US-FDA has approved 51 nanomedicines, with another 77 in the pre-clinical/clinical stage. Some of the commercially available nanomedicines are liposomes, proteins, polymers, micelles, emulsions, nanocapsules, and dendrimers (Mozafari, 2018). Nanotechnology offers numerous possibilities for disease perception, precaution, treatment, and preservation. The potential of nanotechnology in health care and medicine has yet to be completely explored, therefore research and development are required to aid people with serious medical illnesses such as cancer, cardiovascular disease, and chronic kidney disease (Bhardwaj and Burgess, 2010; Ma et al., 2020). We summarize the drug delivery system of nanoparticles in the treatment of various diseases (Table 2). The sizes of the nanoparticles were varying with the disease based on targeting receptor.

**TABLE 2:** Elucidate drug delivery system of nanoparticles in treatment of various disease.

Nanoparticles	Drug	Particle size (nm)	Diseases	Experimental model	References
Chitosan (CS)	Mertansine (MRT), cabazitaxel (CBZ)	112, 110	*Cancer*	Human breast cancer cells (MDA-MB-231, MDA-MB-468, and MCF7)	Pandya et al. (2020)
CS	Curcumin (CUR)	115		Human lung cancer cells (A549), human colon cancer cells (HCT116)	Almutairi et al. (2020)
CS	Boswellic acid (BA)	67.5–187.2		A549 cells	Salanki et al. (2020)
CS	Piperlongumine (PL)	200		Human dermal fibroblast cells (hDFB), human prostate cancer cells (PC-3)	Choi et al. (2019)
CS	Adriamycin (ADM)	86.8–102.5		Kunming (KM) mice	Kou et al. (2017)
Poly (lactide-co-glycolide) (PLGA)	Ferulic Acid (FA)	200		Human lung cancer cells (NCI-H460)	Merlin et al. (2012)
PLGA	Doxorubicin (DOX)	94		BDF1mice, Wistar rats, and *Chinchilla* rabbits	Pereverzeva et al. (2019)
PLGA	Thymoquinone (TQ)	147.2		Human melanoma cells (A375)	Ibrahim et al. (2020)
PLGA	Ursolic acid (UA), caffeine (Caf)	120, 100		Human colon cancer cells (HT29)	Merlin et al. (2017)
PLGA	Brucine (BRU)	94–253		MDA tumor bearing mice	Elsewedy et al. (2020)
PLGA	Paclitaxel (PTX)	127.3		Nude mice	Duan et al. (2019)
				Cellosaurus cell (LO2), MDA-MB-23 cells	
PLGA	R-flurbiprofen (FLUR)	150–190		Wistar rats, rat glioma 2 cells (RG2)	Caban-Toktas et al. (2020)
Poly-lactic acid (PLA)	Docetaxel (DTX)	100		ALB/c nude mice, human liver cancer cells (Hep-G2)	Zhu et al. (2016)
Gold (Au)	DTX	75.90		BALB/c nude	Gotov et al. (2018)
				Mice, human cervical cancer cells (HeLa), MCF-7 cells	
Au	DOX	22		Balb/c mice	Elbialy et al. (2015)
Au	Dichloro (1,2-diaminocyclohexane)platinum (II) (DACHPt)	183		BALB/c nude mice	Lee and Shieh, (2020)
Au	Salinomycin (Sal)	18		MCF-7 cells	Zhao et al. (2019)
Silver (Ag)	Imatinib (imab)	105–210		MCF-7 cells	Shandiz et al. (2017)
Ag	Indocyanine green (ICG)	100		Athymic nude mice, murine melanoma cells (B16F10)	Park et al. (2020)
Physalis mottle virus (PhMV)	Cisplatin	30		NCr nude mice, MDA-MB-231 cells	Hu and Steinmetz, (2020)
PLGA	Insulin	297.8	Diabetes mellitus	Sprague Dawley (SD) rats	Abdelkader et al. (2018)
PLGA	Cinaciguat (CCG)	80		Rat-derived mesangial cells (rMCs)	Fleischmann et al. (2021)
PLGA	Quercetin (Qu)	179.9		SD rats	Chitkara et al. (2012)
PLGA	γ-oryzanol	214.8		*ob*/*ob* mice	Kozuka et al. (2017)
PLA	Lutein	152.38		Wistar albino rats	Mishra et al. (2015)
Mannosylated sodium alginate (MAN-ALG)	Ins2_9−23_ (peptide)	200–300		NOD/ShiLtJNju mice	Qu et al. (2021)
Mesoporous silica (mSiO_2_)	Cerium (III) chloride (CeCl_3_)	87.6		Wistar rats	(Yang et al., 2017)
Ag	Nimesulide (NIM)	64.25		SD rats	(Wang Y et al., 2021)
CS	FA	119.5		Wistar albino rats	Panwar et al. (2018)
CS	CUR	50		Hyperglycaemic rats	Akolade et al. (2017)
CS	Qu	91.58		Wistar rats	Mukhopadhyay et al. (2018)
Solid lipid (SLNs)	Resveratrol (RES)	248		Wistar rats	Mohseni et al. (2019)
SLNs	Berberine (BBR)	76.8		SD rats	Xue et al. (2013)
SLNs	Myricitrin	50–100		NMRI mice	Ahangarpour et al. (2018)
Liposomes	Betanin	40.06		Wistar rats	Amjadi et al. (2019)
Phytosomes	BBR	165.2		Wistar rats, human intestinal epithelial cells (Caco-2)	(Yu F et al., 2016)
Nanocrystals	CUR	32		Albino rats	Gouda et al. (2019)
Nanosuspensions	UA	246.4		Albino Wistar rats	Singh et al. (2019)
Nanosuspensions	BBR	73.1		C57BL/6 mice	Wang et al. (2015)
Nanosuspensions	Betulin	110		SD rats	Zhao et al. (2014)
PLGA	Wogonin (Wog)	194.82	Cardiovascular	Albino Wistar rats	Bei et al. (2020)
PLGA	Vascular endothelial growth factor (VEGF)	113		NOD/SCID mice	Oduk et al. (2018)
PLGA	Insulin-like growth factor (IGF1)	75		FVB mice	Chang et al. (2013)
PLGA	Cyclosporine A (CsA)	100		C57BL/6J, Cyclophilin D−/− mice	Ikeda et al. (2016)
PLGA	Irbesartan	200		C57BL/6J, CCR2^−/−^ mice	Nakano et al. (2016)
PLGA	Pitavastatin	160		SD rats	Nagaoka et al. (2015)
PLGA	Pitavastatin	160		C57BL/6J mice	Mao et al. (2017)
PLA	CUR	96.67		Wistar rats	Li et al. (2021)
SLNs	Daidzein	126		SD rats, Beagle dogs	Gao et al. (2008)
SLNs	Candesartan cilexetil (CC)	180–220		Wistar rats	Dudhipala and Veerabrahma, (2016)
Dendrimer	microRNA-1 inhibitor	50		C57BL/6J mice	Xue et al. (2018)
Liposomes	None	101.5		Swiss mice	Paulis et al. (2012)
Silicon	siRNA, CCR2, MSCs	100–200		BALB/c male mice	Lu et al. (2015)
Polyketal (PK3)	Nox2-siRNA	500		C57BL/6J mice	Somasuntharam et al. (2013)
PK3	Nox2-siRNA	500		C57BL/6J mice	(Yang et al., 2017)
Micelles	Nitroxyl radical	40		Dog, I/R	Asanuma et al. (2017)
Micelles	CCR2 inhibitor	34.7		C57BL/6J mice	(Wang et al., 2018)
Lipid	Cyclosporine A (CsA)	160		Chinese Bama swine	Yin et al. (2014)
Lipid	Puerarin	110		Wistar rats	Dong et al. (2017)
Lipid	Schisandrin B (Sch B)	130		SD rats	Shao et al. (2017)
Iron oxide microrods (MRs)	Tissue plasminogen activator (tPA)	15	Neurogenetic	CD1-IGS mouse	Hu et al. (2018)
PLGA	Estradiol	138.8		SD rats	Mittal et al. (2011)
PLGA	Donepezil	89.63		Wistar rats	Md et al. (2014)
PLGA	Rotigotine	70,000		SD rats	Wang et al. (2012)
PLGA	Antioxidants	250–270		SD rats	Petro et al. (2016)
PLGA	Trimethylated chitosan (TMC)	136.8		APP/PS1 double transgenic mice	Wang et al. (2010)
PLGA	Temozolomide (TMZ)	125.1		SD rats	Chu et al. (2018)
PLGA	Ropinirole (RP)	100–120		Wistar rats	Negro et al. (2017)
CS	Galantamine hydrobromide	48.3–68.3		Wistar rats	Hanafy et al. (2016)
CS	Selegiline	165–255		SD rats	Sridhar et al. (2018)
CS	Polyamidoamine (PAMAM)	197		Wistar rats	Sharma et al. (2018)
CS	Levodopa	Unknown		SD rats	Cao et al. (2016)
CS	Selenium (Se)	33.11		Wistar rats	Asl et al. (2021)
Au	Glutathione (GS)	2.5–3.3	Kidney	UUO mice	(Yu et al., 2016)
Iron oxide (IO)	Gadolinium (Gd)	4.8		BALB/c mice	Zhou et al. (2013)
Carbon dot (C-dot)	Infrared dye (ZW800)	3		Nude mice	Huang et al. (2013)
Palladium (Pd II)	Poly (vinylpyrrolidone) (PVP)	4.4		BALB/c mice	Tang et al. (2014)
Copper (Cu)	GS	2		BALB/c mice	Yang et al. (2015)
Silica (Si)	Anti-CD11b	100		UUO mice	Shirai et al. (2012)
Au	Methoxy-PEG-thio	10		BALB/c mice	Choi et al. (2011)
Gadolinium (AGuIX)	Rhodamine B	3		BALB/c mice	Sancey et al. (2015)
Carboxymethyl dextran, PAMAM G5 dendrimer	None	5		C57BL/6J mice	Nair et al. (2015)
PLGA	Polyethylene glycol (PEG)	400		SKH-1 Elite hairless mice	Williams et al. (2015)
Carbon nanotubes (CNTs)	siRNA	5		BALB/c mice	Alidori, et al. (2016)
Liposomes	Anti-E-selectin antibody (Ab_Esel_)	121		C57BL/6J mice	Asgeirsdottir et al. (2008)
Collagen IV	Anti-inflammatory peptide (Ac2-26)	77.15		C57BL/6J mice	Kamaly et al. (2013)
Polycation (CDP/AD-PEG)	siRNA	60–100		BALB/c mice	Zuckerman et al. (2012)
Albumin	Celastrol	75		SD rats	Guo et al. (2017)
Poly (ethylene glycol)-poly (l-lysine) (PEG-PLL)	siRNA	10		MRL/lpr mice	Shimizu et al. (2010)
Lysozyme	Sunitinib analog (17,864)	Unknown		C57BL/6J mice	Dolman et al. (2012)
Captopril (CAP)	G3-C12 (GSG)	215		Kunming strain mice	Geng et al. (2012)
CS	Rhodamine B	10–200		C57BL/6J mice	(Wang et al., 2021)

### Diagnosis of Kidney Disease by Nanoparticles

A successful intervention therapy could be used to prevent CKD and decrease consequences including infection, hypertension, anemia, and heart failure. On the other hand, traditional diagnostic approaches have several drawbacks, including being inconsiderate and inconvenient. (Ma et al., 2020). Albuminuria is a risk factor for both incident and progressive CKD (Bobo et al., 2016). If the concentration of urinary albumin was greater than 30 mg/dl, it shows positive results with the routine urine analysis dipstick. Microalbuminuria can be detected using specific urinary albumin dipsticks or specific antibody methods (Reichel et al., 2020). Surface-enhanced Raman scattering (SERS) with NPs is the commercially accessible emission technology, which involves the inelastic scattering concerning incident laser energy. It undergoes great sensitivity, simple sample processing, and rapid analysis (Mosier-Boss, 2017). According to one report, the detection of urine albumin levels does not need pre-processing of the samples and the method was quite quick (McNay et al., 2011). Commercial instruments are currently available that allow for microalbuminuria screening at the point of care (Mosier-Boss, 2017). The instruments have been proven beneficial for diagnosing early diabetic kidney disease by comparing the results with devices and laboratory tested, and it could be utilized in remote underdeveloped countries in the future (McNay et al., 2011). Cystatin C (CysC), N-acetyl-d-glucosaminidase (NAG), and kidney injury molecule-1 (KIM-1) are found to be effective predictors of CKD (Kumar et al., 2018). An increasing amount of evidence suggests that NPs could be employed to boost an immunosensor in response to the observation of these indicators (Wang et al., 2016; Lopes et al., 2019). KIM-1 is a biomarker of pre-stage renal injury and indicates the action of renal injury and its recovery has also been determined using an electrochemiluminescence (ECL) biosensor. The electron transfer efficiency was then improved by using platinum (Pt) NPs (Zhang et al., 2018). GFR is a measurement of kidney function, and physicians use the CKD-EPI as a formula to evaluate GFR and are referred to as eGFR (Yang et al., 2018). Furthermore, tubular cells can absorb and secrete a small amount of creatinine, which has been found to be ineffective to detect the CKD in the pre-or post-stages, whereas fluorescent NPs such as qdots, gold, and silica NPs have been found to be providing advantages over current methods to detect the GFR (Takeda et al., 2002).

Fluorescent semiconductor nanocrystals, which are widely used in the biological area and are commercially available (Huang and Gretz, 2017). Moreover, near-infrared fluorescence imaging, which is highly sensitive and affordable, has been widely used in the investigation of various disorders (Wang et al., 2019). Non-invasive fluorescence imaging of renal insufficiency and staging are still in the pre-clinical stage of development (Penna et al., 2011). To measure kidney function, Yu et al. employed the renal clearable near infrared-emitting glutathione-coated gold NPs (GS-AuNPs) as a contrast agent in fluorescence imaging of kidneys. They discovered that this nanotechnology may be used to noninvasively measure kidney disease, describe the stages of malfunction even disclose an adaptive function in mice with unilateral obstructive nephropathy (UUO) (Yu et al., 2016). Furthermore, they were successful in identifying kidney malfunction that corresponded to renal damage as determined by pathological results [134]. Many renal-clearable NPs such as AuNPs, copper NPs (CuNPs), iron oxide NPs, silica NPs (SiNPs), carbon dots, and palladium nanosheets, are now available, allowing NPs to be used in noninvasive renal imaging (Shirai et al., 2012; Huang et al., 2013; Zhou et al., 2013; Tang et al., 2014; Yang et al., 2015). In animal models of UUO, SiNPs with the fluorescent probe anti-CD11b have been used as an imaging tool for measuring inflammation and fibrosis at a high intensity (Shirai et al., 2012). On the other hand, nano-magnetic resonance imaging (nano-MRI) could overcome the limits of traditional modalities and represent the next step in renal biopsy (Khosroshahi et al., 2017). Furthermore, it is non-invasive and examines the status of kidneys, which makes it a useful follow-up test. Iron oxide NPs is possibly an alternative to nephrotoxic gadolinium-based MRI contrast agent in the development of nanoscale sensing technologies (Stabi Bendz, 2011). Before and after 24 h injection of iron oxide, the signal strength of each kidney was assessed by several conditions of nephropathies. In rats with nephrotoxic nephritis, the cortical signal intensity reduced considerably. In the obstructive nephropathy model, the signal intensity is decreased in renal compartments, which is responsible for the diffuse interstitial lesions, suggesting that nano-MRI could aid in the diagnosis of various nephropathies (Hauger et al., 2000; Stabi Bendz, 2011). Nephropathies could be recognized and discriminated against by evaluating the strength and area of an MRI signal. Though the long-term safety of iron oxide NPs is uncertain, their preclinical pharmacokinetics and safety appear to be suitable in terms of application possibilities in a single dose of a diagnostic agent for human MRI (Hauger et al., 2000).

### Treatment of Kidney Disease by Nanoparticles

Renal function is lost as a result of CKD, and the condition can progress to renal failure. Unlike more acute inflammatory glomerulonephritis, though immunosuppression may be curable, there are currently no treatments available to reverse the loss of renal function in CKD (Bourrinet et al., 2006). To date, there are just a few therapeutic options for slowing the progression of CKD. Angiotensin-converting enzyme inhibitors (ACEIs) or angiotensin receptor blockers (ARBs), as well as other conservative medicines, are examples of supportive care (Breyer and Susztak, 2016). NPs are significant because they serve as a kidney-targeted transport route for various medicines and nucleic acids (Ma et al., 2020). The characterized NPs target the ligands that can be tailored to target specific cells or tissues in NPs (Ma et al., 2020). The size of NPs has a big impact on cellular absorption, circulation half-life, and targeting (Sharma et al., 2011). The half-life of NPs with 100 nm is longer than the NPs with smaller or larger diameters. Fewer particles were more likely to pass through the kidneys, although those smaller than 10 nm are more likely to be eliminated via renal excretion and phagocytosis (Sharma et al., 2011).

It has been reported that the larger NPs (>100 nm) were unable to penetrate the renal mesangium due to its size limitation imposed by fenestrations of glomerular endothelial cells (GECs) (Hoshyar et al., 2016). Targeting the renal tubule has also been studied, with NPs (less than 10 nm) engineered to pass through the glomerular filtration barrier and internalized by the epithelial cells (Choi et al., 2011; Sancey et al., 2015). Another study revealed that 5 nm sizes of dextran-based NPs and dendrimer NPs are filtered and absorbed by tubular epithelial cells in a time-or dose-dependent manner *in vivo* (Nair et al., 2015). NPs with 400 nm, which were significantly larger than the glomerular basement membrane’s (GBM) fenestrations, were discovered to selectively target proximal tubules. The data revealed that the NPs are internalized at the basal side by proximal tubule epithelial cells through peritubular capillaries [143]. NPs must be properly delivered and bonded to their specified target, and the shape of NPs has a substantial impact on their performance and biological distribution *in vivo*.

Several shapes have been constructed like cubic, spherical, hexagonal, helical, and rod; even the cylindrical shape is altered by blood flow (Blanco et al., 2015). When compared to other shapes, the rod structure has shown to be more effective at penetrating tumors (Chauhan et al., 2011). Because of the aspect ratio and dimension, spherical NPs clear more slowly than non-spherical NPs; yet they migrate to vessel walls less efficiently (Gentile et al., 2008).

A study suggested that carbon nanotubes are delivered therapeutic siRNA to proximal tubular cells reducing injury in cisplatin-induced kidney injury models (Alidori, et al., 2016). Coating liposomes with an anti-E-selectin antibody effectively suppressed E-selectin expression in GECs and reduced albuminuria (Asgeirsdottir et al., 2008). NPs carrying the anti-inflammatory peptide Ac2-26 have been demonstrated to bind with collagen IV and targets subendothelial collagen IV (Kamaly et al., 2013). Targeting medications of proximal tubular cells may be a promising way for the treatment of tubulointerstitial fibrosis, as it reduces the likelihood of negative side effects while increasing the potency of antifibrotic therapies (Dolman et al., 2012). Megalin is the multi-ligand receptor, which is expressed in the apical membrane of proximal tubular epithelial cells and plays a key function in the cell’s endocytosis (Wicher et al., 2006). Several studies have investigated whether the megalin receptor can transport pharmaceuticals to the kidneys specifically based on targeting active sites (Gao et al., 2014; Oroojalian et al., 2017).

### Toxicity of Nanoparticles

NPs may cause cellular toxicity through oxidative stress, inflammation, and interactions with the cell membrane (Prow, 2010; Occhiutto et al., 2020). On the other hand, nano-toxicity is determined by the purity of the molecule and the drug concentrations, as well as other features such as nanoparticle size, surface form, and ionic charge (Voigt et al., 2014). Particle, excipient, contaminant, and inflammatory toxicity are all examples of toxicity that could be caused by independent drug delivery (De Matteis and Rinaldi, 2018). *In vitro and in vivo* models should be used to study each toxicity separately. There is a lack of specific literature on the toxicity of therapeutic NPs (Prow, 2010). Nano-toxicity is a critical topic that must be investigated in connection with each NP’s therapeutic potential, especially for those that require multiple doses (De Jong et al., 2013; El-Ansary et al., 2013; De Matteis and Rinaldi, 2018). Before receiving full regulatory approval, all nanoparticles that are suggested to be used for therapeutic reasons must be thoroughly examined in terms of local and systemic toxicity.

## Kidney Targeted Nanoparticle Drug Delivery System

The structure of kidneys and features of drug delivery devices has been extensively investigated in order to successfully deliver medications to the kidney (Kamaly et al., 2016; Chen et al., 2020). The delivery strategy of kidney-targeted drug delivery systems might be discussed in detail in the following sections, with emphasis on key targets such as GECs, GBM, podocytes, mesangial cells (MCs), and proximal tubules, which play critical roles in the progressions of kidney diseases (Chen et al., 2020).

### Key Targets of Kidney

GECs, which are found in the glomerular capillary wall, are the first portion of the glomerular filtration barrier. Fenestrations and transcellular pores (60–160 nm) are packed with the endothelial glycocalyx that distinguishes them (Kamaly et al., 2016). GECs and their surface of glycocalyx play a key role in the plasma component filtering (Ichimura et al., 2008). Renal failure, proteinuria, and glomerulosclerosis are all illnesses associated with endothelial filtration abnormalities (Satchell and Braet, 2009). A second glomerular filtration barrier is formed by GBM, a unique extracellular matrix linked to GECs. According to the research, GBM is discovered to be a reticular connective tissue, which is made up of proteoglycan, collagen IV, and the laminin is released by the GECs and the podocytes (Suleiman et al., 2013). Meshes of a diameter of 4–10 nm are found on the surface of GBM, which could become larger in the presence of renal illness (Gagliardini et al., 2010). Heparin sulfate proteoglycan, which has a large negative charge, is the major component of proteoglycans, which causes GBM with negatively charged to produce repulsion for blood and impede plasma albumin filtration (Yu et al., 2019). As a result of the electrostatic adsorption of proteoglycan, the cationic carrier can be employed in drug delivery systems, which might be target GBM. Zuckerman and colleagues found that siRNA nanoparticles made of cationic polymer could reach the kidney quickly. After 6 min of injection, the siRNA signal in GBM had accumulated to roughly 20% of the typical dose (Zuckerman et al., 2012).

Podocytes connected to the outside of the GBM and represent the final barrier of glomerular filtration. The foot processes and slit diaphragm are distinctive structural features of podocytes, with pore sizes ranging from 30 to 40 nm. In between podocyte foot processes, the glomerulus filtration capacity may be controlled, and big molecules in the blood cannot get through, which is important for the kidney’s filtration barrier (Lal et al., 2015). Due to its highly differentiated characteristics and limited ability to repair or regenerate, Podocytes are easily damaged (Brinkkoetter et al., 2013). Podocytes are involved in glomerulosclerosis, glomerulonephritis, membranous nephropathy, and diabetic nephropathy, among other kidney illnesses (Pavenstadt et al., 2003). The upregulation of the integrin αvβ3 receptor in diabetic podocytes could be the target for the medication delivery system (Mallipattu and He, 2016). In a separate study, inorganic nanoparticles treated with an integrin v3 antibody were found to be more selectively taken up by podocytes than unmodified inorganic nanoparticles (Pollinger et al., 2012). On the other hand, the preceding examples of targeted podocytes are all done out *in vitro* in cell models. The drugs must have a diameter of at least 10 nm in order to pass through GECs and GBM and reach podocytes *in vivo*. FcRn receptors were discovered on podocytes, and they were determined to be responsible for IgG internalization (Akilesh et al., 2008). MCs are responsible for expanding and balancing the glomerular matrix, regulating the filtration surface area, and eliminating immune complexes, as well as maintaining the glomerular microvascular bed’s structural integrity (Wu et al., 2017). As a result, medications administered to MCs for the treatment of numerous renal illnesses are beneficial (Zhao et al., 2019). Nanoparticles with smaller sizes than GEC pores (60–160 nm) can be delivered to the MCs due to the lack of GBM and podocytes on the side of the glomerular capillary closest to the MCs (Scindia et al., 2008). As a result, the size of the nanoparticle is a critical component in establishing a direct link between the mesangial region and circulation. Choi and colleagues found that the gold nanoparticles with 75 ± 25 nm diameter accumulated successfully in the mesangial area of mice, which indicates that nanoparticles with a diameter of 50–100 nm could be effective in targeting MCs (Choi et al., 2011). Another study found that the combination of albumin with celastrol NPs to target MCs could pass through fenestrated endothelium, accumulate in MCs, and improve renal pathological morphology in nephritis model rats with the results showing that the albumin-celastrol NPs with 95 nm size could pass through fenestrated endothelium and accumulate in MCs, which improve renal pathological morphology (Guo et al., 2017). Furthermore, Shimizu et al. discovered that the mitogen-activated protein kinase 1 (MAPK1) siRNA NPs with 10–20 nm size delivered by poly (ethylene glycol) (PEG)-poly (l-lysine) copolymer-based delivery vehicles successfully penetrated the pores of GECs into the mesangial region, decreased MAPK1 mRNA, TGF-1, and fibronectin expression (Shimizu et al., 2010). Targeted alteration happens according to the particle size, which can improve nanoparticle uptake by MCs, which express the mannose receptor in the kidney, which can mediate nanoparticle uptake *in vivo* study (Zhang et al., 2011).

Proximal tubular cells, which are responsible for the active movement of endogenous and foreign substances between blood and urine, are the most active cells in renal physiological metabolism. They can boost the tubulointerstitial inflammatory response, as well as the development and progression of fibrosis, by presenting antigens and producing cytokines (Ramos et al., 2015). As a result, medication delivery to the proximal tubular cells is extremely beneficial for lowering tubulointerstitial fibrosis, inflammation, and boosting renal tubular regeneration (Falke et al., 2015). The ability of drug delivery methods to reach proximal tubular cells is largely determined by their characteristics and the anatomical structure of the kidney. The drug delivery methods can reach proximal tubular cells in two ways. The apical side of the proximal tubule is the first place to look for proximal tubular cells (Kaissling et al., 1996). To reach the proximal tubular cells, drug delivery methods must have a particle size of at least 10 nm. The second approach to get to proximal tubular cells is through the proximal tubule’s basolateral side (Kaissling et al., 1996). To begin, the particles must pass via the renal peritubular capillaries, which contain endothelial fenestrations measuring 60–70 nm in diameter (Satchell and Braet, 2009). A diaphragm with 3–5 nm thickness is roughly closed these fenestrations (Nico et al., 2009). The study demonstrated that the positively charged particles can easily pass through the fenestrations because they contain negatively charged heparin sulfate (Satchell and Braet, 2009).

### Potential Drug Carriers to Proximal Tubules

**FIGURE 1:**
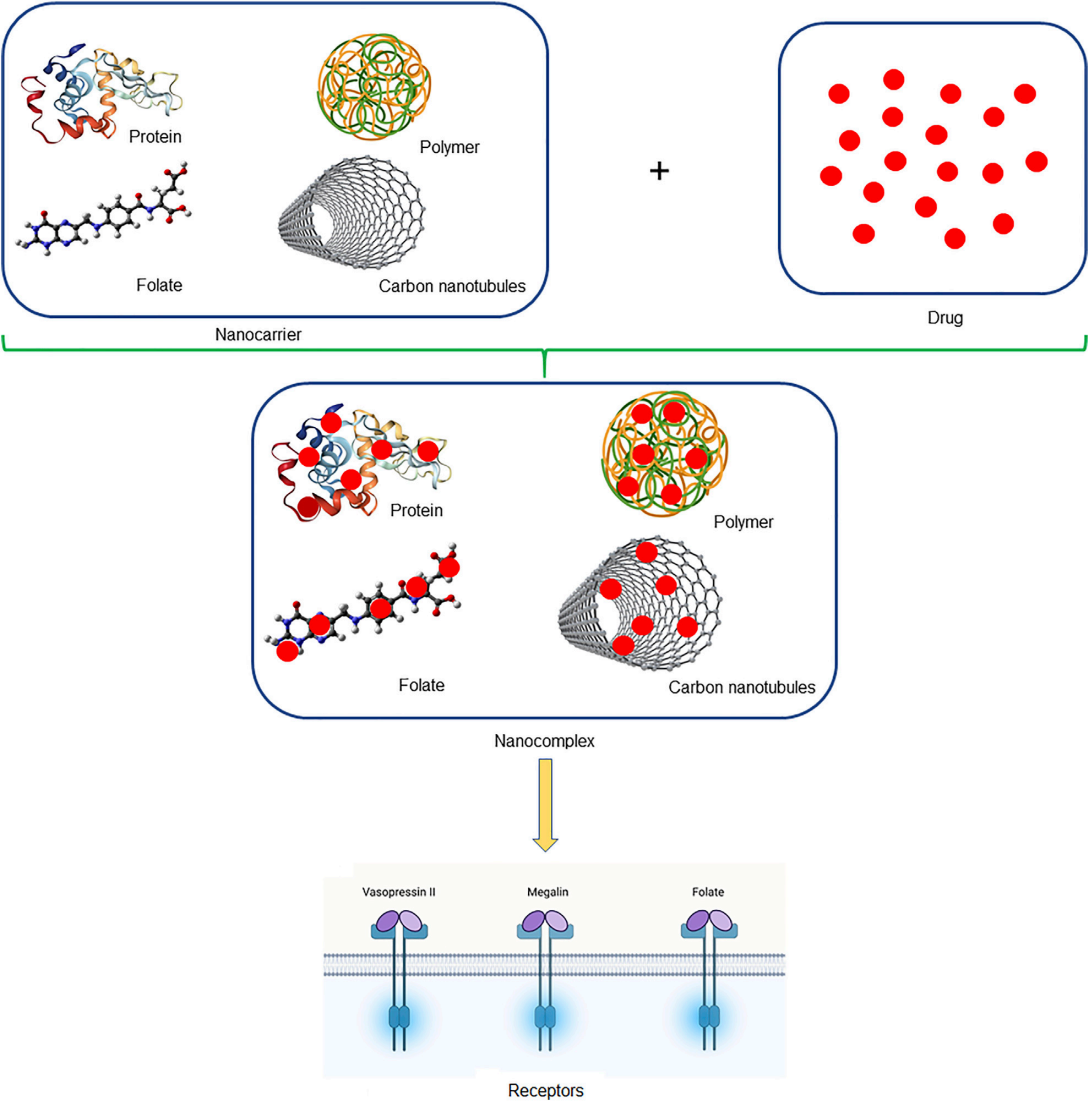
Schematic illustration of nanocomplex. The figure mentioned briefly the nanocarriers which were used to carry drugs in the form of nanocomplex, which tends to deliver drugs safely to the kidney.

We summarize the potential drug carriers to proximal tubules (Figure 1), and some advantages and disadvantages of nanocarriers are shown in Table 3. Low molecular weight proteins (LMWP) have been extensively studied for delivering drugs to proximal tubular cells. The molecular weight and charge of LMWP determine its capacity to permeate the glomerular filtration barrier and the degree of reabsorption by the proximal tubular cells. Lysozyme (LMZ) is the best researched LMWP protein with a molecular weight of 14 KDa and the capacity to flow nearly easily past the glomerular filtration barrier before being reabsorbed by the megalin receptors of proximal tubular cells, lysozyme (LMZ) is the best researched LMWP protein (Christensen et al., 2009).

**TABLE 3:** Advantages and disadvantages of nanocarriers.

Nanocarriers	Advantages	Disadvantages
Polymeric nanoparticles	Drug release in controlled and sustained manner	Difficulty for their scale-up
	Incorporation of hydrophilic and hydrophobic drugs	Insufficient of toxicological assessment in the literature
	Use of a lot of biodegradable materials when desired	
	Higher stability than lipid-based ones	
	Multiple functional groups for targeted drug delivery	
	Covalently associating drugs	
	Protecting drug from environmental conditions	
	Reproducible data when used synthetic polymers	
	Higher stability than lipid-based ones	
	Being many methods to prepare them	
	Existence of pH, enzymatic, hydrolysis, etc., sensitive properties when preferred proper polymers	
	Tunable chemical and physical properties	
	Acting like solubility enhancers	
Lipid-based nanoparticles (SLNs)	Producing on large industrial scale	Low drug loading for SLNs
	Low toxicity due to their biocompatible and biodegradable components and absence of organic solvent(s) in their process	Risk of gelation for SLNs
	Incorporation of lipophilic and hydrophilic drugs	Drug expulsion during storage cause by lipid polymorphism for SLNs
	Protecting drug from environmental conditions	Limited penetration in the skin
	Low cost compared with liposomes	Skin irritation due to high concentration of alcohol for ethosomes
	Protecting drug from environmental conditions	Lack of robust controlled drug release
	High encapsulation efficiency	Loss of high amounts of drug
	Low toxicity	Restricted transdermal drug delivery
	Enhanced penetration and permeation	
	Good stability during storage period	
	Possibility of specific follicular targeting	
	Avoid systemic absorption and side effects in dermal drug delivery Purpose	
	Easy and scalable production process	
	Improving oral bioavailability	
	Long physical stability	
	Longer drug circulation time	
	Preventing undesired plasma peak	
Metal nanoparticles	Desired size and shape nanoparticle can be prepared	High energy required
	Most effective method for less volatile raw materials	Extensive long period of milling time
	Relatively simple and effective technique for the formation of large number of small particles (nano-size) in the form of suspension	Contamination of powder due to steel balls
	Nanoparticles formation is possible without adding surfactant in liquid media	Very sensitive microstructure can be grinded
	Less impurities are generated than those created by chemical methods	Generates low volume of material
	Well-crystallized powder can be formed	
	Particle size can be controlled by modifying flow rate of chemicals through the pyrolysis reaction zone	
	Relatively simple method and low cost	
	Produce nanocrystal with high crystallinity	
	Chemical vapor deposition method of coating exhibits the high film durability	
	Simple method for the formation of thin metal films	

By connecting lysozyme to a sunitinib analog, Dolman et al. created the kidney-targeted conjugated LMZ-17864 particles (Dolman et al., 2012). After a single dose, LMZ-17864 immediately accumulated in the kidney, which persisted for 3 days. When compared to free sunitinib, the drug level in the kidneys was boosted 28 times and no clear harmful side effects were seen. These conjugates can target the kidney efficiently and quickly, halting the progression of kidney disease (Zhang et al., 2009). Peptides could be employed as a drug carrier to target proximal tubular cells in addition to LMWP (Wang et al., 2017). Both kidneys had a 25 and 46% accumulation content of radiolabeled gastrin and glucagonlike peptide 1 (Gotthardt et al., 2007). According to Geng et al., a combination of G3-C12 peptide and a small molecule model medication captopril (CAP) to form G3-C12-captopril (G3-C12-CAP) (Geng et al., 2012). Fluorescence imaging revealed that G3-C12-CAP accumulated swiftly and precisely in the kidneys of mice, owing to proximal tubular cell reabsorption. G3-C12-CAP demonstrated a 2.7-fold increase in the kidney when compared to free CAP. MCLPVAS is a seven-amino-acid peptide that targets the kidneys (Geng et al., 2012). An elastin-like polypeptide (ELP) is a non-immunogenic protein transporter, which can connect tiny molecules and peptides in a stable manner (Cho et al., 2016).

Anionic polyvinylpyrrolidone (PVP) has been shown to be a potential kidney-targeted carrier. Approximately 30% of the administered dose accumulated in the kidneys when mice were given carboxylated PVP intravenously for 3 h (Kodaira et al., 2004). Liu et al. created the panel of 25-kDa p (OEGMA-co-MAA) copolymers made up of different ratios of neutral methyl glycol methacrylate (OEGMA) to anionic methacrylic acid (MAA) (1:0, 1:1, 1:4). The polymers are accumulated in the kidney, and the amount accumulated increases as the quantity of anionic monomer (Liu et al., 2018). According to Yamamoto et al., polyvinylpyrrolidone-co-dimethyl maleic acid (PVD) is a drug carrier with strong kidney-targeting ability and safety. Renal accumulation of PVD with the molecular weight of 6–8 kDa has the highest renal level (Yamamoto et al., 2004). As a kidney-targeted drug carrier, Kamada and colleagues created polyvinylpyrrolidone-co-dimethyl maleic anhydride (PVDn). At high doses, roughly 80% of PVDn was selectively deposited in the kidney after 24 h of intravenous administration to mice and did not produce kidney or other tissue damage (Kamada et al., 2003). Low molecular weight chitosan (LMWC) is a biocompatible and degradable polymer that can be utilized by targeting kidneys (He et al., 2012). Yuan et al. used a chemical reaction to link 50% of the N-acetylated LMWC with a molecular weight of 19 kDa to the model medication prednisolone (Yuan et al., 2007). In mice, 14% of LMWC conjugates accumulated in the kidney within 15 min of treatment, and the prednisone levels are boosted by 13 times in the kidney. Liang et al. formulated AZT-COS by the combination of chitosan oligomers (COS) and zidovudine (AZT). The average retention period of AZT-COS in the body is raised by 2.5 times, and the cumulative amount in the kidney is increased by 5.6 times when compared to free AZT (Liang et al., 2012). Yuan and others found that LMWC uptake was considerably reduced in megalin-shedding animals, implying that the LMWC might be entered into the proximal tubular cells via the megalin receptor (Yuan et al., 2009). Wang et al. have investigated that chitosan nanoparticles (CS-NP) promising as drug delivery platforms for oral delivery in kidney disease (Wang J et al., 2021).

The antibodies with a higher molecular weight of around 150 kDa cannot pass into the glomerular filtration barrier and are challenging to drug carriers for proximal tubular cell targeting (Akizawa et al., 2008). According to the study, the antibody fragments with less than 50 kDa molecular weight can pass through the glomerular filtration barrier and reabsorb by the proximal tubular cells. Radiolabeled monoclonal antibody fragments could clump together in the proximal tubules of the kidney (Grunberg et al., 2005). As a result, targeting proximal tubular cells with antibody fragments as a drug carrier could be a realistic strategy.

A prodrug is a drug derivative that can be digested or activated at a specific target site, allowing active medicines to be released or produced (Kratz et al., 2008). Prodrugs-based folate and glycosyl have been shown to target tubules in the studies. Folate is a nutrient that is often used in the delivery of anti-cancer drugs (Cheng et al., 2017; Abazari et al., 2019). Folate can be used as a ligand to transport drugs to proximal tubular cells because the folate receptor on these cells can be reabsorbed (Mathias et al., 2000). After a 5-min intravenous administration of the radiolabeled DTPA-folate conjugate [99mTc]DTPA-folate in mice. The radiolabeled DTPA-folate conjugate [99mTc]DTPA-folate was quickly absorbed by the kidney, according to Mathias et al. The kidney collected roughly 21% of the injected dose per Gram tissue after 4 h of intravenous administration. The level of [99mTc]DTPA-folate in the kidney reduced to 2.3 percent when free folic acid was pre-injected (Trump et al., 2002). Similar investigations found that after intravenous injection of [99mTc](CO)3-DTPA-folate for 4 h, the accumulation in the kidney is approximately 47% of the injected dose per Gram tissue (Trump et al., 2002). As a knowledge, folate could be the effective vehicle for drug delivery to the kidneys.

Carbon nanotubes (CNTs) are the new delivery vehicle in medicinal and diagnostic applications due to their unique intrinsic physical, chemical, and optical capabilities. According to McDevitt, CNTs with a length of 100–500 nm and a diameter of 1–2 nm are filtered through the glomerulus and reabsorbed in the proximal tubule (McDevitt et al., 2007). After a 20-min injection of CNTs in mice, around 65% of CNTs were eliminated by the kidney and about 15% were reabsorbed in the proximal tubules, even though CNTs were much larger than glomerular filtration (Ruggiero et al., 2010). The study discovered that CNT-mediated siRNA was preferentially delivered to proximal tubular cells and successfully shut off the expression of multiple target genes to minimize kidney injury in model mice with acute renal injury (Alidori et al., 2016). Another study found that CNTs might pass through the glomerulus and enter the renal capsule in a direction perpendicular to the GBM (Lacerda et al., 2008).

### Mechanism of Targeted Drug Delivery in Kidney

The clearance rate of medications and therapeutic materials from the body is one of the most important elements influencing their pharmacokinetics. NPs enter the systemic circulation after absorption, disperse and interact with the body before being removed by the reticuloendothelial system (RES) or the kidney (Moss and Siccardi, 2014). The kidneys receive 1–1.2 L of blood per minute or 20–25% of cardiac output. The basic functional unit of the kidney, the nephron, has an average of one million nephrons in each kidney. The renal corpuscle, or glomerulus, as well as the proximal and distal tubules, make up each nephron. The primary filtrate is transmitted through fenestrated capillaries into the Bowman’s capsule, which encloses the glomerulus and collects the filtrate, via the renal vasculature to the glomeruli. The filtrate is subsequently passed via the proximal and distal renal tubules, where nutrients, water, and ions are reabsorbed, and waste products are released (Hauser et al., 2021).

**FIGURE 2:**
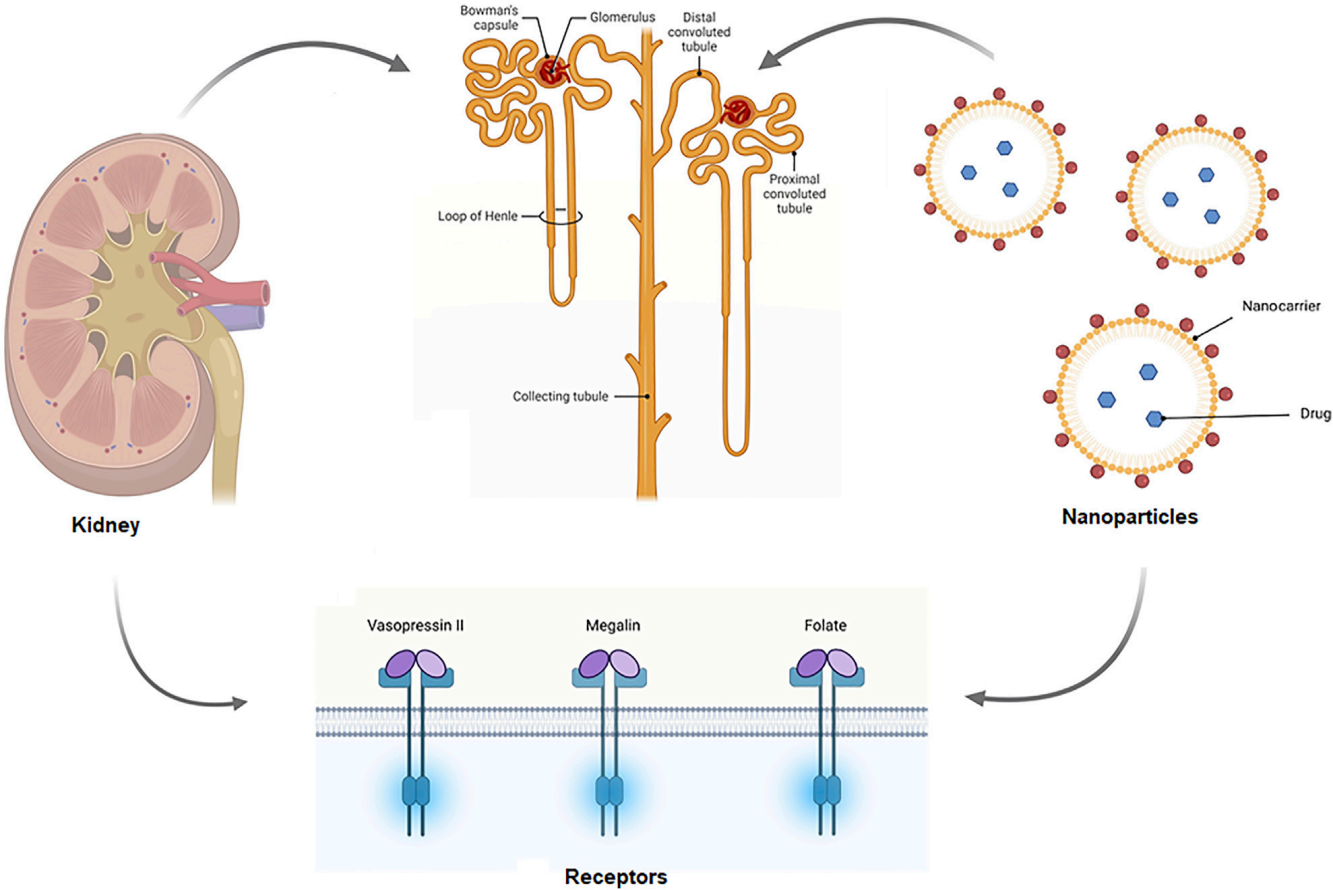
Schematic of the mechanism of targeted drug delivery in the kidney. Drug carrying nanoparticles enter the kidney via the renal artery and are carried to the afferent arteriole, where they remain in the bloodstream or are subjected to renal filtration from the blood in the glomerular capillaries. Renal components such as the glycocalyx, endothelial cells, and the glomerular basement membrane can be tailored to help select NPs for filtration. NPs can interact with podocytes in the Bowman’s lumen after filtering. The filtrates NPs are carried to the proximal tubule, where they interact with proximal epithelial cells and might be reabsorbed.

When constructing nanomedicine, the size, shape, and charge of nanoparticles must be considered, since these will determine whether they are cleared or accumulated by the kidneys. A positively charged nanomedicine, for example, may be able to filter through a negatively charged GBM and podocytes more efficiently than neutral or negatively charged substances (Hauser et al., 2021). Interaction sites and probable uptake sites of NPs, which are extracted or employed to target the kidney. NPs enter the kidney via the renal artery and are carried to the afferent arteriole, where they remain in the bloodstream or are subjected to renal filtration from the blood in the glomerular capillaries, depending on particle properties. Renal components such as the glycocalyx, endothelial cells, and the glomerular basement membrane can be tailored to help select NPs for filtration is shown in Figure 2. NPs can interact with podocytes in the Bowman’s lumen after filtering. The filtrates NPs are carried to the proximal tubule, where they interact with proximal epithelial cells and may be reabsorbed. Pro-drug NPs can be triggered in proximal tubular cells lysosomes. After being delivered from the efferent arteriole to the peritubular network, NPs that are not selected for renal filtration can interact with the renal tubular compartment (Hauser et al., 2021). Targeting was done in two ways in the drug delivery system, which are active and passive targeting. Active targets used the ligand-receptor approach to locate the ultimate target, whereas passive targeting uses the enhanced permeability (EPR) effect to locate specific spots of the receptor (Hossen et al., 2019). The vasopressin II receptor (V2R) is a G protein-coupled receptor (GPCR) with seven transmembrane domains that is expressed in the basolateral membrane of epithelial cells that line the distal tubule, connecting tubule, and collecting ducts (Robben et al., 2004), which might be useful for targeted drug delivery through the active target. In renal fibrosis, megalin receptor-mediated endosomal absorption of various medicines and safe release of the active drug from endosomes (Nastase et al., 2018). Although characterized NPs might allow for passive accumulation in the kidneys, active targeting nanoparticles, such as peptides and antibodies, are also being investigated to increase kidney targeting (Huang et al., 2021).

## Conclusion and Future Perspectives

Kidney disease is becoming a major epidemiologic problem over the world. Despite significant research achievements, the pathophysiologic processes involved in the progression of many kidney disorders are still unknown. Some nanomaterials have been used in clinical therapy as a result of the rapid development of nanomedicine. The goal of creating kidney-targeted drugs is to increase medication levels and therapeutic efficacy while reducing drug toxicity and side effects. Altering the characteristic features of nanoparticles of the drug delivery system is based on the physiological or pathological characteristics of the kidney can help achieve the goal of targeting the kidney. Although nanotechnology has shown some benefits in kidney illness, there are several difficulties to be handled and improved, including limited manufactured products, expensive, need *in vivo* steadiness, less selective regulation, and possible lesions to nontarget organs. The current focus of research on kidney-targeted drug delivery systems is on finding acceptable porters and boosting targeted productivity when the release profile and the metabolic processes of drug delivery systems after they penetrate targeted cells are still being studied. Based on the knowledge, our future research will focus on building nanoparticles containing therapeutic drug-based kidney-targeted drug delivery systems.

## Abbreviations

Ag, Silver; Au, Gold; CKD, Chronic kidney disease; CS, Chitosan; DLS, Dynamic light scattering; FDA, Food and Drug Administration; FT-IR, Fourier-transform infrared spectroscopy; GBM, Glomerular basement membrane; GECs, Glomerular endothelial cells; MCs; Mesangial cells; NPs, Nanoparticles; PLGA, Poly (lactide-co-glycolide); TEM, Transmission electron microscopy; UV-Vis, Ultraviolet-visible spectroscopy; XRD, X-ray diffraction.
